# Impact of macrolide treatment on long-term mortality in patients admitted to the ICU due to CAP: a targeted maximum likelihood estimation and survival analysis

**DOI:** 10.1186/s13054-023-04466-x

**Published:** 2023-05-31

**Authors:** Luis Felipe Reyes, Esteban Garcia, Elsa D. Ibáñez-Prada, Cristian C. Serrano-Mayorga, Yuli V. Fuentes, Alejandro Rodríguez, Gerard Moreno, Alirio Bastidas, Josep Gómez, Angélica Gonzalez, Christopher R. Frei, Leo Anthony Celi, Ignacio Martin-Loeches, Grant Waterer

**Affiliations:** 1 Universidad de La Sabana, Campus Puente del Común, KM 7.5 Autopista Norte de Bogotá, Chía, Colombia; 2 Clínica Universidad de La Sabana, Chía, Colombia; 3 University of Oxford, Oxford, UK; 4 Hospital Universitari Joan XXIII, Critical Care Medicine, Rovira and Virgili University and CIBERES (Biomedical Research Network of Respiratory Disease), Tarragona, Spain; 5 College of Pharmacy, The University of Texas at Austin, San Antonio, TX USA; 6 School of Medicine, University of Texas Health San Antonio, San Antonio, TX USA; 7 Massachusetts Institute of Technology, Cambridge, USA; 8 Beth Israel Deaconess Medical Center, Boston, USA; 9 Harvard T.H. Chan School of Public Health, Boston, USA; 10 Department of Intensive Care Medicine, Multidisciplinary Intensive Care Research Organisation (MICRO), St. James’s Hospital, Dublin, Ireland; 11 Royal Perth Bentley Hospital Group, University of Western Australia, Perth, Australia

**Keywords:** Community-acquired pneumonia, Macrolide, Mortality, β-lactam

## Abstract

**Introduction:**

Patients with community-acquired pneumonia (CAP) admitted to the intensive care unit (ICU) have high mortality rates during the acute infection and up to ten years thereafter. Recommendations from international CAP guidelines include macrolide-based treatment. However, there is no data on the long-term outcomes of this recommendation. Therefore, we aimed to determine the impact of macrolide-based therapy on long-term mortality in this population.

**Methods:**

Registered patients in the MIMIC-IV database 16 years or older and admitted to the ICU due to CAP were included. Multivariate analysis, targeted maximum likelihood estimation (TMLE) to simulate a randomised controlled trial, and survival analyses were conducted to test the effect of macrolide-based treatment on mortality six-month (6 m) and twelve-month (12 m) after hospital admission. A sensitivity analysis was performed excluding patients with *Pseudomonas aeruginosa* or MRSA pneumonia to control for Healthcare-Associated Pneumonia (HCAP).

**Results:**

3775 patients were included, and 1154 were treated with a macrolide-based treatment. The non-macrolide-based group had worse long-term clinical outcomes, represented by 6 m [31.5 (363/1154) vs 39.5 (1035/2621), *p* < 0.001] and 12 m mortality [39.0 (450/1154) vs 45.7 (1198/2621), *p* < 0.001]. The main risk factors associated with long-term mortality were Charlson comorbidity index, SAPS II, septic shock, and respiratory failure. Macrolide-based treatment reduced the risk of dying at 6 m [HR (95% CI) 0.69 (0.60, 0.78), *p* < 0.001] and 12 m [0.72 (0.64, 0.81), *p* < 0.001]. After TMLE, the protective effect continued with an additive effect estimate of − 0.069.

**Conclusion:**

Macrolide-based treatment reduced the hazard risk of long-term mortality by almost one-third. This effect remains after simulating an RCT with TMLE and the sensitivity analysis for the HCAP classification.

**Supplementary Information:**

The online version contains supplementary material available at 10.1186/s13054-023-04466-x.

## Introduction

Community-acquired pneumonia (CAP) is a leading cause of infectious death worldwide and one of the principal causes of admission to the Intensive Care Unit (ICU) [1, 2]; its annual worldwide mortality varies between 50,000 and 100,000 patients [3], and the overall hospital mortality associated with CAP varies from 20 to 50% [4]. Notably, mortality due to CAP in the ICU has remained steady during the last decade, impacting healthcare systems tremendously worldwide. Its economic burden exceeds $17 billion in the United States and more than €10 billion annually in Europe [5]. While the main focus of research has been the acute illness in CAP patients, survivors have a significantly higher mortality risk for up to ten years after the acute episode [6, 7]. It has been suggested that long-term mortality (i.e., after hospital discharge) in CAP patients may be related to a chronic proinflammatory state documented after hospital discharge [8, 9]. However, the specific mechanism underlying long-term mortality in CAP patients remains unclear, although excess cardiac mortality is highly likely to be one contributing cause [10–12].

Treatment for patients with CAP admitted to the ICU is based on broad-spectrum antibiotics and early goal-directed therapy [13]. Some studies have suggested that using at least two antibiotics with different mechanisms of action is associated with better acute clinical outcomes [14, 15]. One study in elderly patients hospitalised with CAP demonstrated several cardiovascular benefits, except for myocardial infarction, in patients who received azithromycin plus another appropriate antibiotic [16]. Recent data have shown that using β-lactams plus macrolides might reduce the length of hospital stay and mortality in patients with CAP admitted to the ICU [17]. The proposed mechanisms to explain the beneficial effect of including a macrolide in treating patients with CAP (i.e., macrolide-based treatment) [18, 19] are the atypical coverage and the anti-inflammatory effect produced by inhibiting intracellular signalling pathways such as the NFkB [8, 20]. Some researchers have hypothesised that macrolides might improve clinical outcomes by decreasing the production and liberation of some toxins produced by Gram-positive bacteria, such as the pneumolysin produced by the *Streptococcus pneumoniae* and the Panton-Valentine Leucocidin, produced by the *Staphylococcus aureus* [21–24].

The Infectious Diseases Society of America and American Thoracic Society (IDSA/ATS) guidelines recommend using macrolide-based treatment in patients admitted to the ICU due to CAP [13]. However, data exploring the long-term implications of this recommendation is lacking. We hypothesise that patients treated with macrolide-based treatment have lower long-term mortality [i.e., six-month (6 m) and twelve-month (12 m) mortality]. To test this hypothesis, we performed a multivariate analysis, targeted maximum likelihood estimation (TMLE), and survival analysis of patients admitted to the ICU due to CAP using the Medical Information Mart for Intensive Care IV (MIMIC-IV) database, a large prospective cohort of patients hospitalised in the ICU.

## Material and methods

This is a prospective cohort of patients admitted to the ICU and registered to the MIMIC-IV database. Registries were taken from the multi-parametric intelligent monitoring data from the ICU at the Beth Israel Deaconess Medical Centre (BIDMC) in Boston, Massachusetts, containing the complete information of 69,639 patients admitted to the ICU between 2008 and 2019 (https://doi.org/10.13026/7vcr-e114). The Laboratory of Computational Physiology (LCP) created the database at the Massachusetts Institute of Technology (MIT). The database is supported by the National Institute of Biomedical Imaging and Bioengineering (NIBIB) of the National Institutes of Health (NIH) [25]. Further information about the database can be found elsewhere (https://lcp.mit.edu/mimic).

### Participants

**Fig. 1:**
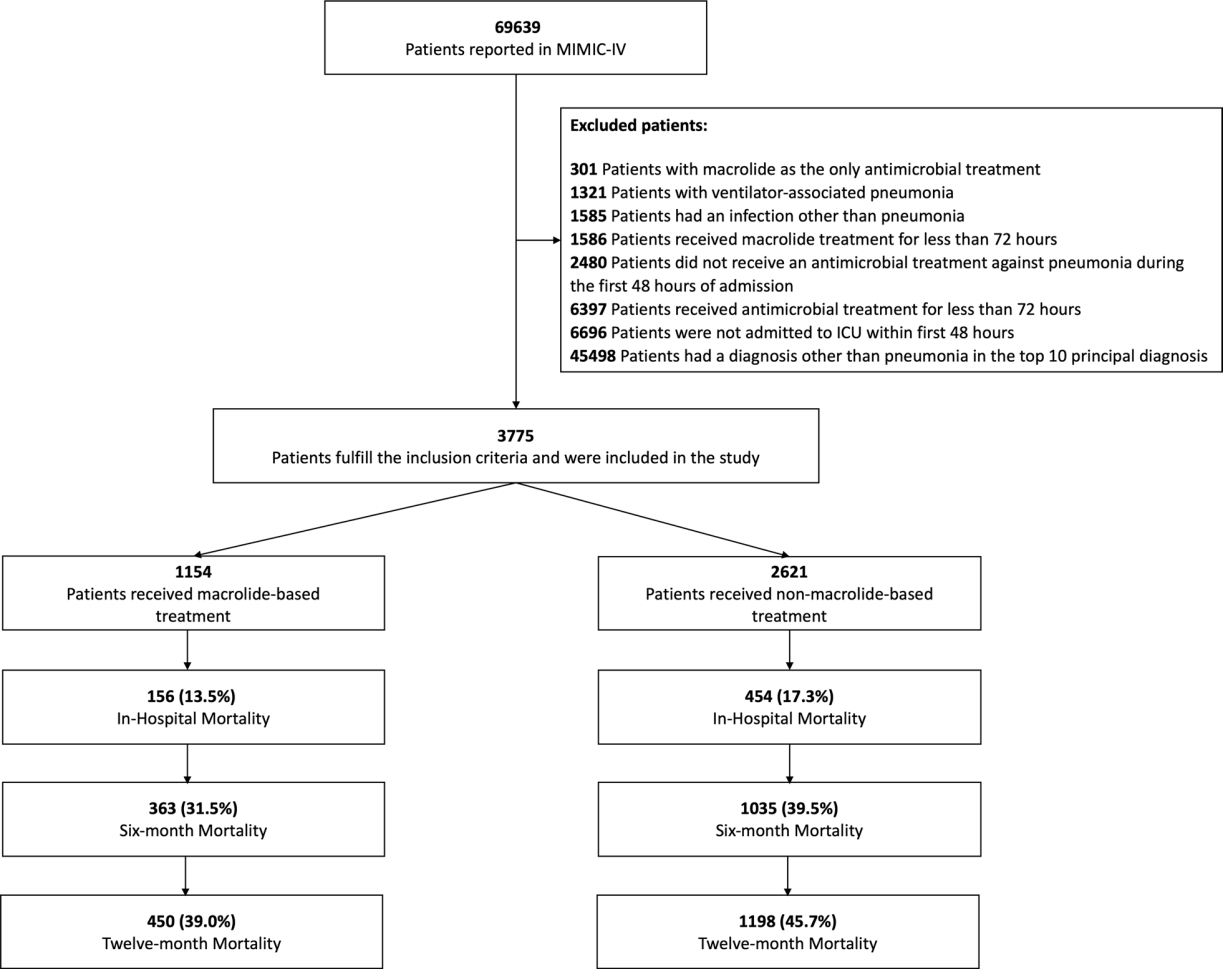
Study flow chart of patients included in the analysis

The cohort included patients admitted to the ICU due to CAP. The definition of CAP was based on the ATS/IDSA guidelines [13]. The inclusion criteria were patients older than 16 requiring admission to the ICU with an ICD-9 code of pneumonia within the top ten diagnoses and must receive pneumonia-related antibiotics during the first 48 h of hospital admission. The MIMIC IV database organises diagnoses by cost, not by relevance. All the included diagnoses are shown in Additional file 1: Table S1. Patients with another infectious diagnosis different to pneumonia during the first 48 h, transferred patients, those in whom antibiotic treatment was suspended during the first 96 h, those who received a macrolide for less than 48 h, and with less than 70% of the numerical data (labs and vital signs) were excluded from the study (Fig. 1).

### Study groups

The cohort was stratified depending on the antibiotic administered during the first 48 h of admission: macrolide-based and non-macrolide-based treatment. Patients who received an antibiotic accepted by international guidelines plus a macrolide (e.g., azithromycin or clarithromycin) were included in the macrolide-based treatment group. In contrast, those who received other antibiotics recommended by the IDSA/ATS guidelines but not a macrolide were included in the non-macrolide-based treatment group [13, 26]. Notably, all patients were treated with at least a β-lactam (including carbapenems) or a fluoroquinolone.

### Data collection

The following variables were obtained during the first 24 h of admission: demographic data, comorbidities (i.e., Charlson comorbidity index), urine output, laboratory data, invasive interventions, severity (i.e., Simplified Acute Physiology Score II [SAPS II]), and outcomes. The data was taken directly from the critical care information systems, electronic hospital records, laboratory results, and vital signs monitors, as described before [25]. All information was secured with read-only access to ensure data integrity. The MIMIC-IV database is integrated with the US Social Security System to allow access to mortality data even after hospital discharge [25].

### Statistical analysis

Continuous variables were described as minimum or maximum values, mean and standard deviation (SD), or median and interquartile range (IQR), depending on their clinical relevance and distribution. Dichotomous variables were presented as frequencies and percentages. For the univariate analysis, differences between the intervention groups were assessed with the chi-square test and Fisher's exact test for categorical variables or the student's t-test or Mann–Whitney U test for continuous variables, depending on their distribution.

A multivariate logistic regression model was developed in the general cohort to evaluate the relationship between 6 and 12 m mortality (dependent variable) and demographics, comorbid conditions, and laboratory variables (explanatory variables). The logistic regression model included variables with a *p* < 0.20 in the initial univariate analysis. The fitness of the model was assessed with the Hosmer–Lemeshow test. Odds ratios (OR) were calculated based on the exponentials of the coefficients obtained by the final model and presented in forest plots. To evaluate the performance of the logistic regression model, the area under the model's receiver operating curve (AUROC) was used; for this, a tenfold cross-validation method was used, in which the dataset was divided into ten subsets, and the validation was repeated ten times. Each time, one of the subsets was used as the test cohort, and the other nine subsets were used as training subsets, and then the average AUROC was calculated and reported. Also, a Cox Proportional Hazard Model was constructed to evaluate the hazard ratios (HR) for mortality at 6 m and 12 m. This model was adjusted by demographics, comorbid conditions, and laboratory variables. Adjusted HR were calculated and presented in tables.

Finally, we performed a targeted maximum likelihood estimation (TMLE) analysis to simulate a randomised controlled trial using the baseline characteristics to estimate the one-year mortality average effect of macrolide-based treatment in critical patients with CAP admitted to the ICU. TMLE is used for the estimation of causal effects using observational data. TMLE estimates both the outcome and the treatment mechanisms and requires an initial estimate of the conditional expectation of the outcome given the exposure, the covariates, and the probability of being treated given the observed confounders, which is done using ensemble and machine-learning algorithms. Then, it performs a substitution step that optimises the bias-variance trade-off for the average treatment effect (ATE). TMLE calculates the adjusted marginal difference in mean outcome associated with a binary point treatment for continuous or binary outcomes. To estimate the ATE of macrolide treatment, we calculated the marginal risk difference of one-year mortality for patients receiving macrolide-based therapy versus those not receiving it through TMLE. A significance level of 0.05 and a confidence level of 95% were chosen. Data analysis was done using R and SPSS (IBM) version 29.

## Results

A total of 3775 patients were included in the study (Fig. 1). Most patients were male [57.9% (2185/3775)], and the mean (SD) age was 67.6 (6.1) years. Comorbidities were evaluated with the Charlson comorbidity index, with a mean (SD) of 6.5 (3.0) points (Table 1). Also, more than half of the patients developed respiratory failure [58.6% (2213/3775)], and a quarter developed septic shock [26.9% (1017/3775)]. All invasive interventions are shown in Additional file 1: Fig. S1A. Severity was estimated with the SAPS II, with a mean (SD) of 38.5 (13.2) corresponding to a 21.3–23.0% risk of in-hospital mortality during the acute episode. Finally, regarding long-term mortality, almost half of the cohort died within a year of ICU admission due to CAP [43.7% (1648/3775)] (Table 1, Fig. 1).

**Table 1:** Demographic characteristics of all patients and stratified between treatments

Characteristic	All cohort (n = 3775)	Macrolide-based (n = 1154)	Non-macrolide-based (n = 2621)	*p*-value
*Demographic*				
Male, n (%)	2185 (57.9)	642 (55.6)	1543 (58.9)	0.07
Age, mean (SD)	67.6 (16.1)	68.0 (16.2)	67.4 (16.0)	0.19
Charlson comorbidity index, mean (SD)	6.5 (3.0)	6.6 (3.0)	6.5 (3.0)	0.43
*Laboratory variables at admission, mean (SD)*				
Haematocrit min, %	30.6 (6.6)	31.6 (6.7)	30.2 (6.6)	< 0.001
Haemoglobin max, mg/dL	11.3 (2.2)	11.4 (2.3)	11.2 (2.2)	0.06
Platelets min, cell/mm^3^	205.3 (119.6)	200.5 (104.0)	207.4 (125.9)	0.74
WBC min, cell/mm^3^	11.3 (10.0)	11.5 (10.8)	11.3 (9.6)	0.73
WBC max, cell/mm^3^	15.1 (12.6)	15.0 (14.7)	15.1 (11.5)	0.04
Anion gap max, mEq/L	17.2 (4.9)	17.3 (4.6)	17.2 (5.0)	0.11
Bicarbonate min, mEq/L	22.3 (5.6)	22.5 (5.7)	22.3 (5.6)	0.49
BUN max, mg/dL	33.1 (25.3)	32.3 (23.3)	33.4 (26.1)	0.99
Calcium max, mEq/L	8.6 (0.9)	8.6 (0.7)	8.6 (0.9)	0.05
Chloride min, mEq/L	100.1 (6.9)	99.0 (6.9)	100.6 (6.9)	< 0.001
Creatinine min, mEq/L	1.4 (1.4)	1.4 (1.4)	1.4 (1.4)	0.82
Glucose min, mg/dL	121.2 (44.8)	123.3 (46.1)	120.3 (44.1)	0.04
Sodium max, mEq/L	139.7 (5.5)	139.5 (5.4)	139.9 (5.6)	0.30
Potassium max, mEq/L	4.7 (0.9)	4.8 (1.0)	4.7 (0.9)	0.001
Lymphocytes max, cell/mm^3^	1.5 (5.0)	1.5 (6.5)	1.4 (4.1)	0.64
Neutrophils max, cell/mm^3^	11.4 (6.9)	11.2 (7.0)	11.5 (6.9)	0.31
INR max	1.8 (1.3)	1.8 (1.3)	1.7 (1.3)	0.34
PT max, sec	19.0 (13.6)	19.1 (13.5)	19.0 (13.6)	0.06
PTT max, sec	43.9 (28.4)	42.6 (26.4)	44.4 (29.2)	0.67
Urine output, mL	1752.4 (1246.4)	1801.4 (1238.4)	1730.8 (1249.6)	0.05
*Interventions, n (%)*				
HFNC	118 (3.1)	73 (6.3)	45 (1.7)	< 0.001
Invasive ventilation	1481 (39.2)	370 (32.1)	1111 (42.4)	< 0.001
Non-invasive ventilation	151 (4.0)	75 (6.5)	76 (2.9)	< 0.001
*Severity, n (%)*				
SAPS II	38.5 (13.2)	37.2 (12.1)	39.1 (13.5)	< 0.001
Respiratory failure	2213 (58.6)	751 (65.1)	1462 (55.8)	< 0.001
Septic shock	1017 (26.9)	311 (26.9)	706 (26.9)	0.98
ARDS	42 (1.1)	19 (1.6)	23 (0.9)	0.06
*Other antibiotic treatments, n (%)*				
Quinolones	1417 (37.5)	246 (21.3)	1171 (44.7)	< 0.001
*Aetiology, n (%)*				
No aetiology	970 (25.7)	269 (23.3)	701 (26.7)	0.03
Atypical bacteria	276 (7.3)	46 (4.0)	230 (8.8)	< 0.001
Typical bacteria	198 (5.2)	35 (3.0)	163 (6.2)	< 0.001
*P. aeruginosa* or MRSA	533 (14.1)	95 (8.2)	438 (16.7)	< 0.001
Fungi	41 (1.1)	19 (1.6)	22 (0.8)	0.04
Virus	1 (0.0)	0 (0)	1 (0.0)	0.67
*Outcomes, n (%)*				
Hospital mortality	610 (16.2)	156 (13.5)	454 (17.3)	0.004
6 m mortality	1398 (37.0)	363 (31.5)	1035 (39.5)	< 0.001
12 m mortality	1648 (43.7)	450 (39.0)	1198 (45.7)	< 0.001

*SD* Standard derivation; *BUN* Blood urea nitrogen; *WBC* White blood cells; *INR* International normalised ratio; *PT* Prothrombin time; *PTT* Partial thromboplastin time; *HFNC* High flow nasal cannula; *SAPS* Simplified acute physiology score; *ARDS* Acute respiratory distress syndrome

**Fig. 2:**
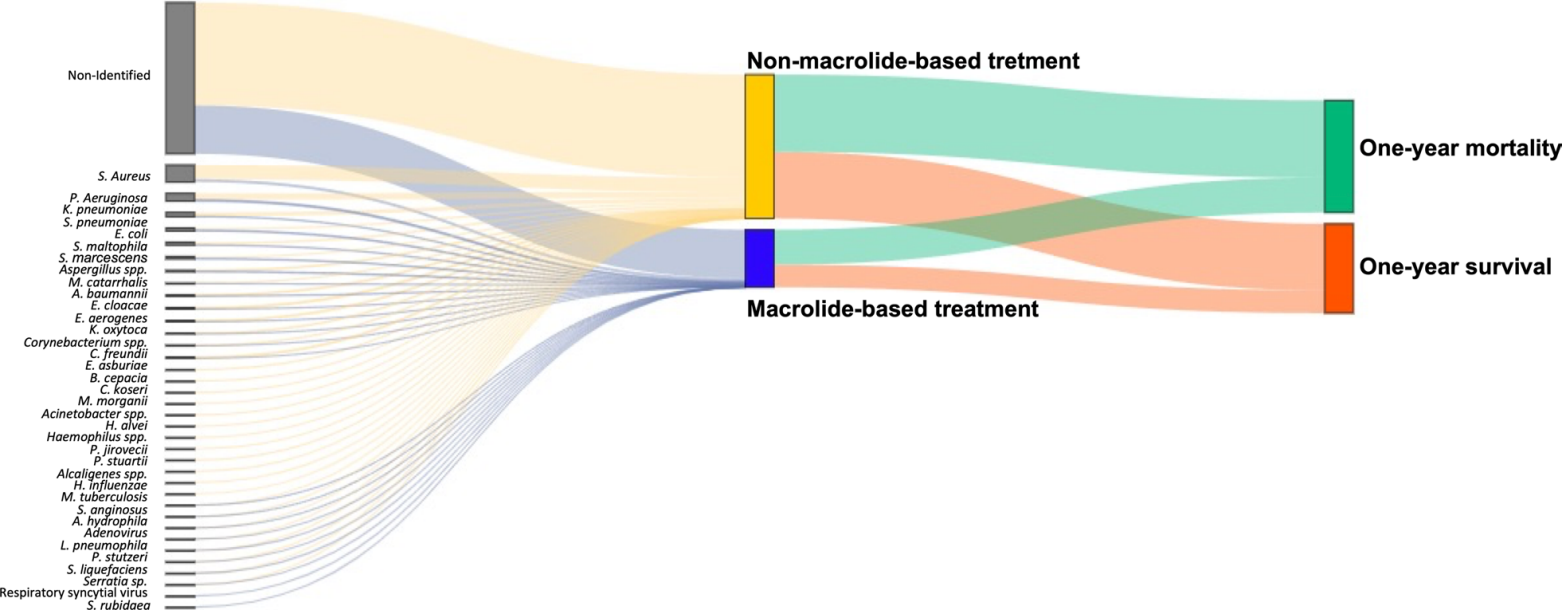
Pneumonia causal agents, treatment received, and long-term outcome. Alluvial diagram of pneumonia causative agents, treatment, and one-year mortality

Only 1127 of the patients had an identified microbiological pathogen. The most frequently identified microorganisms were *Staphylococcus aureus* [34.0% (383/1127)], *Pseudomonas aeruginosa* [15.6% (176/1127)], *Klebsiella pneumoniae* [8.7% (98/1127)], and *Streptococcus pneumoniae* [6.1% (69/1127)] (Additional file 1: Fig. S1B and S1C, Table S2). Regarding antibiotic treatment, most of the patients were treated with vancomycin [47.9% (1809/3775)] and cefepime [26.5% (1001/3775)] (Additional file 1: Table S3). An alluvia present the causative pneumonia agents, treatment, and long-term mortality, as shown in Fig. 2.

A total of 30.6% (1154/3775) received macrolide-based therapy and 69.4% (2621/3775) non-macrolide-based treatment. Both had similar mean (SD) age [68.0 years (16.2) vs 67.4 years (16.0), *p* = 0.19], gender [male: 55.6 (642/1154) vs 58.9 (1543/2621), *p* = 0.07], and mean (SD) Charlson comorbidity index score [6.6 points (3.0) vs 6.5 (3.0), *p* = 0.43]. Patients who received macrolide-based treatment were more often treated with non-invasive ventilatory supports [high flow nasal cannula: 6.3 (73/1154) vs 1.7 (45/2621), *p* < 0.001; non-invasive ventilation: 6.5 (75/1154) vs 2.9 (76/2621), *p* < 0.001]. In contrast, those that received non-macrolide-based treatment more frequently received invasive mechanical ventilation [32.1 (370/1154) vs 42.4 (1111/2621), *p* < 0.001]. Also, the non-macrolide-based group had worse long-term clinical outcomes, represented by 6 m [31.5 (363/1154) vs 39.5 (1035/2621), *p* < 0.001] and 12 m mortality [39.0 (450/1154) vs 45.7 (1198/2621), *p* < 0.001] (Table 1).

### Multivariate analysis between patients with macrolide-based versus non-macrolide-based treatment

**Fig. 3:**
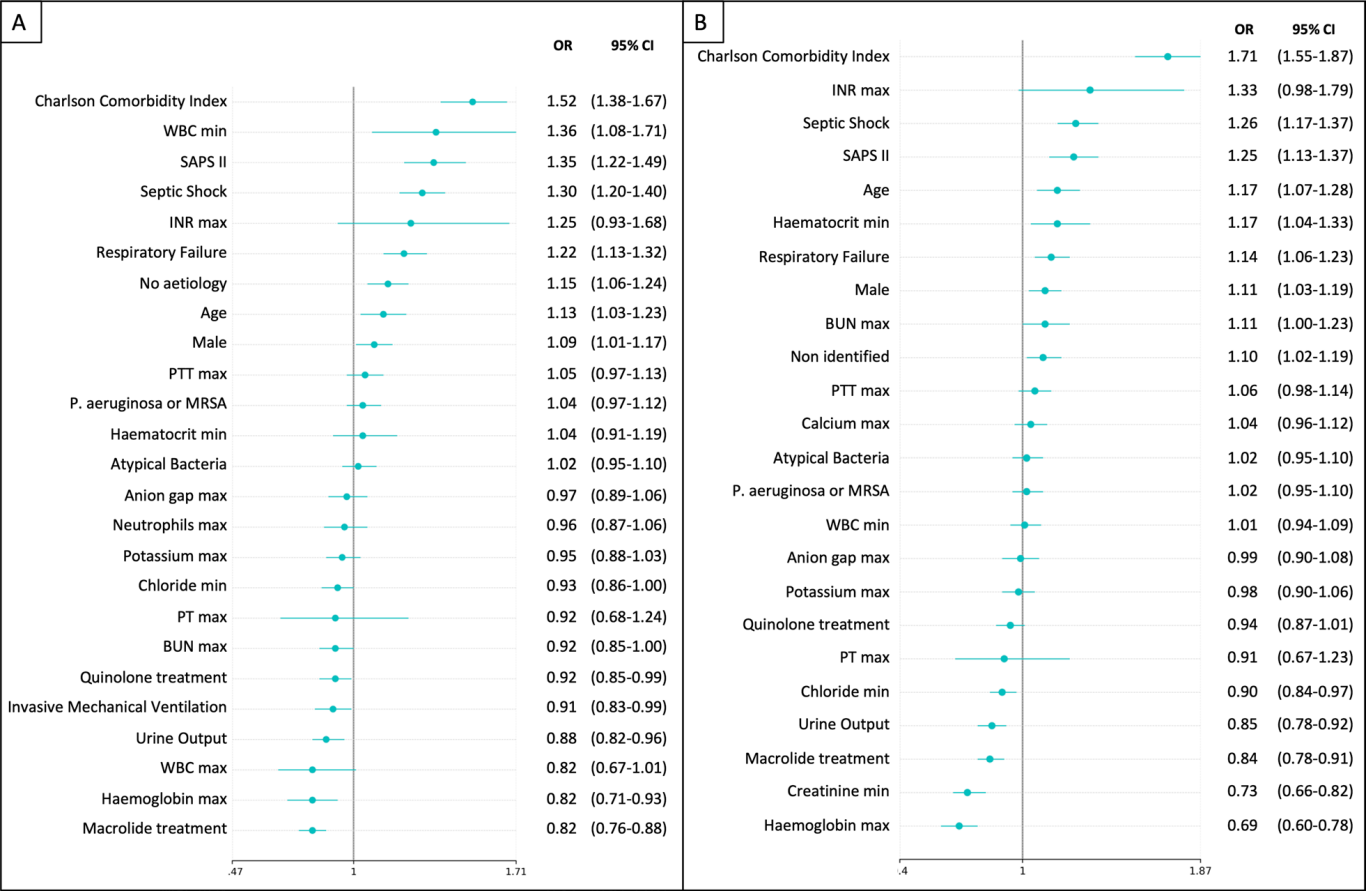
Logistic regression model to identify factors associated with 6 m and 12 m mortality. Logistic regression was performed with the optimal subset of variables obtained with the random forest model. The odds ratios (OR) are graphically represented in the Forest plot for better medical interpretability. Panel **A** presents the odd proportions of the risk for 6 m mortality, and panel **B** shows 12 m mortality

**Fig. 4:**
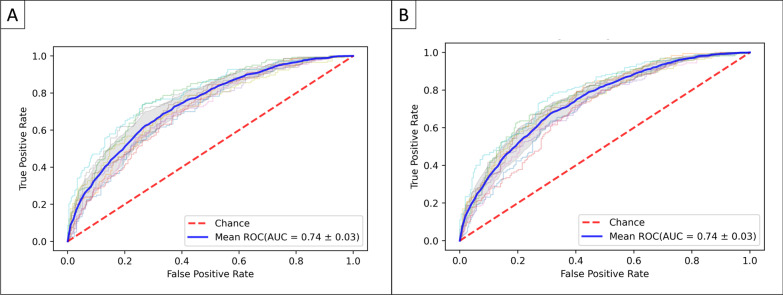
Area under de Curve. Cross-validation trial's receiver operative curve (ROC) for the subset of the selected variables. The blue curve represents the average of the ROC curves of each test, and the average area under de ROC is also presented. Panel **A** shows the AUC-ROC for 6 m mortality and panel **B** for 12 m mortality

After adjusted variables, a logistic regression model was performed for 6 m and 12 m mortality (Tables 2 and 3). The main risk factors associated with mortality were a higher Charlson comorbidity index [6 m: OR (95%CI) 1.52 (1.38–1.67), *p* < 0.001; 12 m: 1.71 (1.55–1.87), *p* < 0.001], SAPS II [6 m: OR (95%CI) 1.35 (1.22–1.49), *p* < 0.001; 12 m: 1.25 (1.13–1.37), *p* < 0.001], septic shock [6 m: OR (95%CI) 1.30 (1.20–1.40), *p* < 0.001; 12 m: 1.26 (1.17–1.37), *p* < 0.001], and respiratory failure [6 m: OR (95% CI) 1.22 (1.13–1.32), *p* < 0.001; 12 m: 1.14 (1.06–1.23), *p* = 0.01]. Moreover, macrolide-based treatment was a protective factor for 6 m and 12 m mortality as compared to patients treated with non-macrolide-based treatment [6 m: OR (95%CI) 0.82 (0.76–0.88), *p* < 0.001; 12 m: 0.84 (0.78–0.91), *p* < 0.001] (Fig. 3A and B). The model used had a good discriminatory capacity when evaluated by the AUROC, mean (SD) of 0.74 (0.03) for 6 m and 12 m (Fig. 4A and B) and appropriate fitness determined by Hosmer Lemeshow Test shown in Additional file 1: Fig. S2.

**Table 2:** Univariate analysis and logistic regression analysis for 6 m mortality

Variable	Univariate analysis		Multivariate analysis	
	OR (95% CI)	*p*-value	OR (95% CI)	*p*-value
*Demographic*				
Male	1.12 (0.98–1.28)	0.10	1.09 (1.01–1.17)	0.02
Age	1.03 (1.02–1.03)	< 0.001	1.13 (1.03–1.23)	0.01
Charlson comorbidity index	1.25 (1.22–1.28)	< 0.001	1.52 (1.38–1.67)	< 0.001
*Laboratory variables at admission*				
Haematocrit min, %	0.96 (0.95–0.97)	< 0.001	1.04 (0.91–1.19)	0.55
Haemoglobin max, mg/dL	0.86 (0.84–0.89)	< 0.001	0.82 (0.71–0.93)	0.01
Platelets min, cell/mm^3^	1.00 (1.00–1.00)	0.91		
WBC min, cell/mm^3^	1.01 (1.00–1.02)	0.01	1.36 (1.08–1.71)	0.01
WBC max, cell/mm^3^	1.01 (1.00–1.01)	0.01	0.82 (0.67–1.01)	0.06
Anion gap max, mEq/L	1.02 (1.01–1.04)	0.01	0.97 (0.89–1.06)	0.5
Bicarbonate min, mEq/L	0.99 (0.98–1.00)	0.21		
BUN max, mg/dL	1.01 (1.01–1.01)	< 0.001	0.92 (0.85–1.00)	0.06
Calcium max, mEq/L	1.05 (0.97–1.13)	0.23		
Chloride min, mEq/L	0.99 (0.98–1.00)	0.13	0.93 (0.86–1.00)	0.04
Creatinine min, mEq/L	1.03 (0.98–1.08)	0.22		
Glucose min, mg/dL	1.00 (1.00–1.00)	0.25		
Sodium max, mEq/L	1.00 (0.99–1.01)	0.91		
Potassium max, mEq/L	1.09 (1.01–1.16)	0.02	0.95 (0.88–1.03)	0.18
Lymphocytes max, cell/mm^3^	1.01 (0.99–1.02)	0.24		
Neutrophils max, cell/mm^3^	1.01 (1.00–1.02)	0.15	0.96 (0.87–1.06)	0.41
INR max	1.20 (1.14–1.27)	< 0.001	1.25 (0.93–1.68)	0.14
PT max, sec	1.02 (1.01–1.02)	< 0.001	0.92 (0.68–1.24)	0.59
PTT max, sec	1.01 (1.00–1.01)	< 0.001	1.05 (0.97–1.13)	0.21
Urine output, mL	1.00 (1.00–1.00)	< 0.001	0.88 (0.82–0.96)	0.01
*Interventions*				
HFNC	1.05 (0.72–1.53)	0.80		
Invasive ventilation	1.18 (1.03–1.35)	0.02	0.91 (0.83–0.99)	0.03
Non-invasive ventilation	1.06 (0.76–1.49)	0.72		
*Severity*				
SAPS II	1.05 (1.04–1.05)	< 0.001	1.35 (1.22–1.49)	< 0.001
Respiratory failure	1.76 (1.54–2.02)	< 0.001	1.22 (1.13–1.32)	< 0.001
Septic shock	2.30 (1.99–2.67)	< 0.001	1.30 (1.20–1.40)	< 0.001
ARDS	1.41 (0.77–2.60)	0.27		
*Antibiotic treatment*				
Macrolide-based	0.70 (0.61–0.81)	< 0.001	0.82 (0.76–0.88)	< 0.001
Quinolones	0.78 (0.65–0.93)	0.01	0.92 (0.85–0.99)	0.03
*Aetiology*				
No aetiology	1.57 (1.36–1.83)	< 0.001	1.15 (1.06–1.24)	0.01
Atypical bacteria	1.36 (1.06–1.74)	0.02	1.02 (0.95–1.10)	0.58
Typical bacteria	1.06 (0.79–1.43)	0.69		
*P. aeruginosa* or MRSA	1.25 (1.04–1.51)	0.02	1.04 (0.97–1.12)	0.30
Fungi	1.21 (0.65–2.25)	0.56		
Virus	0.00 (0.00–0.00)	1.00		

*BUN* Blood urea nitrogen; *WBC* White blood cells; *INR* International normalised ratio; *PT* Prothrombin time; *PTT* Partial thromboplastin time; *HFNC* High flow nasal cannula; *SAPS* Simplified acute physiology score; *ARDS* Acute respiratory distress syndrome

**Table 3:** Univariate analysis and logistic regression analysis for 12 m mortality

Variable	Univariate analysis		Multivariate analysis	
	OR (95% CI)	*p*-value	OR (95% CI)	*p*-value
*Demographic*				
Male	1.10 (0.97–1.26)	0.14	1.11 (1.03–1.19)	0.01
Age	1.03 (1.03–1.04)	< 0.001	1.17 (1.07–1.28)	0.01
Charlson comorbidity index	1.28 (1.25–1.31)	< 0.001	1.71 (1.55–1.87)	< 0.001
*Laboratory variables at admission*				
Haematocrit min, %	0.96 (0.95–0.97)	< 0.001	1.17 (1.04–1.33)	0.01
Haemoglobin max, mg/dL	0.85 (0.83–0.88)	< 0.001	0.69 (0.60–0.78)	< 0.001
Platelets min, cell/mm^3^	1.00 (1.00–1.00)	0.84		
WBC min, cell/mm^3^	1.01 (1.00–1.01)	0.04	1.01 (0.94–1.09)	0.76
WBC max, cell/mm^3^	1.00 (1.00–1.01)	0.24		
Anion gap max, mEq/L	1.02 (1.01–1.03)	0.01	0.99 (0.90–1.08)	0.77
Bicarbonate min, mEq/L	1.00 (0.99–1.01)	0.9		
BUN max, mg/dL	1.01 (1.01–1.01)	< 0.001	1.11 (1.00–1.23)	0.06
Calcium max, mEq/L	1.06 (0.99–1.15)	0.10	1.04 (0.96–1.12)	0.38
Chloride min, mEq/L	0.99 (0.98–1.00)	0.04	0.90 (0.84–0.97)	0.007
Creatinine min, mEq/L	1.03 (0.98–1.08)	0.18	0.73 (0.66–0.82)	< 0.001
Glucose min, mg/dL	1.00 (1.00–1.00)	0.50		
Sodium max, mEq/L	1.00 (0.99–1.01)	0.95		
Potassium max, mEq/L	1.11 (1.04–1.19)	0.01	0.98 (0.90–1.06)	0.56
Lymphocytes max, cell/mm^3^	1.01 (0.99–1.02)	0.42		
Neutrophils max, cell/mm^3^	1.00 (0.99–1.01)	0.58		
INR max	1.24 (1.17–1.31)	< 0.001	1.33 (0.98–1.79)	0.06
PT max, sec	1.02 (1.01–1.02)	< 0.001	0.91 (0.67–1.23)	0.53
PTT max, sec	1.01 (1.00–1.01)	< 0.001	1.06 (0.98–1.14)	0.16
Urine output, mL	1.00 (1.00–1.00)	< 0.001	0.85 (0.78–0.92)	< 0.001
*Interventions*				
HFNC	0.98 (0.68–1.42)	0.92		
Invasive ventilation	1.05 (0.92–1.20)	0.48		
Non-invasive ventilation	1.09 (0.79–1.51)	0.61		
*Severity*				
SAPS II	1.05 (1.04–1.05)	< 0.001	1.25 (1.13–1.37)	< 0.001
Respiratory failure	1.59 (1.39–1.81)	< 0.001	1.14 (1.06–1.23)	0.01
Septic shock	2.10 (1.81–2.43)	< 0.001	1.26 (1.17–1.37)	< 0.001
ARDS	1.18 (0.64–2.16)	0.60		
*Antibiotic treatment*				
Macrolide-based	0.76 (0.66–0.88)	< 0.001	0.84 (0.78–0.91)	< 0.001
Quinolones	0.81 (0.69–0.96)	0.02	0.94 (0.87–1.01)	0.10
*Aetiology*				
No aetiology	1.40 (1.21–1.62)	< 0.001	1.10 (1.02–1.19)	0.02
Atypical bacteria	1.28 (1.00–1.63)	0.05	1.02 (0.95–1.10)	0.52
Typical bacteria	0.93 (0.69–1.24)	0.61		
*P. aeruginosa* or MRSA	1.17 (0.97–1.40)	0.10	1.02 (0.95–1.10)	0.56
Fungi	1.12 (0.60–2.07)	0.72		
Virus	0.0 (0.00–0.00)	1.00		

*BUN* Blood urea nitrogen; *WBC* White blood cells; *INR* International normalised ratio; *PT* Prothrombin time; *PTT* Partial thromboplastin time; *HFNC* High flow nasal cannula; *SAPS* Simplified acute physiology score; *ARDS* Acute respiratory distress syndrome

A propensity score matching (PSM) was performed according to the patient's clinical characteristics and CURB 65 score (Additional file 1: Fig. S3) as a sensitivity analysis. Results showed that macrolide treatment continues to be a protective factor against 12 m mortality [OR (95% CI) 0.85 (0.77–0.94)] (Additional file 1: Fig. S4), with an AUROC 0.71 (0.04) (Additional file 1: Fig. S5).

### Survival analysis

**Fig. 5:**
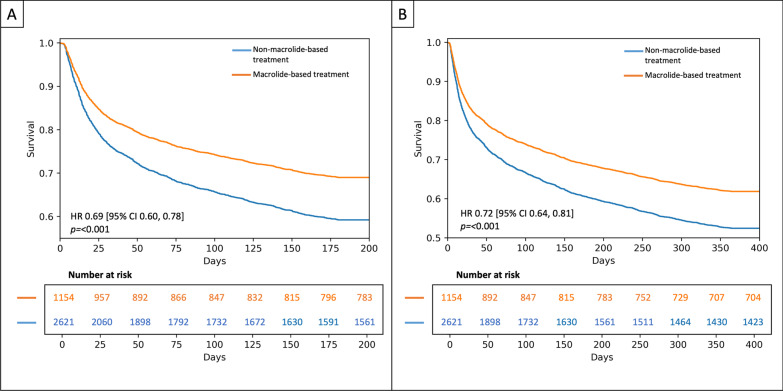
Survival models. Cox Proportional Hazard Curves to identify factors associated with **A** 6 m mortality and **B** 12 m mortality

Cox Proportional Hazard Model analysis (Fig. 5) identified a lower adjusted risk for 6 m and 12 m mortality when patients were treated with macrolide-based treatment [6 m: HR (95% CI) 0.69 (0.60, 0.78), *p* < 0.001; 12 m: 0.72 (0.64, 0.81), *p* < 0.001] compared to non-macrolide-based. The Cox Proportional Hazard Regression output is shown in Additional file 1: Fig. S6, Tables S4 and S5.

### Targeted maximum likelihood estimation (TMLE) analysis

After TMLE analysis, the association of macrolide treatment with one-year mortality remained significant (*p* < 0.001). The Additive Effect (AE) and 95% CI estimates were − 0.059 (− 0.081, − 0.036). Our simulation of a randomised controlled trial using the TMLE analysis suggested positive associations between macrolide-based treatment with a significant reduction of mortality risk in patients admitted to the ICU due to CAP.

### Sensitivity analysis

Healthcare-associated pneumonia (HCAP) was a classification proposed to identify patients at higher risk of developing CAP due to *P. aeruginosa* or methicillin-resistant *S. aureus* (MRSA). Due to its low clinical utility, this classification was removed from the current ATS/IDSA guidelines. However, many doctors have used it for many years. Thus, we performed a sensitivity analysis excluding patients with confirmed *P. aeruginosa* or MRSA CAP and demonstrated the protective value of the macrolide-based treatment even after excluding these patients (Additional file 1: Figs. S7 and S8).

## Discussion

This study found that almost half of the patients admitted to the ICU due to CAP died within one year after the acute episode. Additionally, patients with a higher Charlson comorbidity index, SAPS II, septic shock, and respiratory failure had a higher probability of dying within one year of the acute CAP hospitalisation in the ICU. After a comprehensive statistical analysis of this large prospective cohort, our results suggest that macrolide-based treatment reduces long-term mortality in patients admitted to the ICU due to CAP. Although results regarding coverage of atypic microorganisms with fluoroquinolones proved to be a protector factor at 6 m, this was a vague association that was not maintained thereafter.

Different studies have shown that long-term morbidity and mortality rates in CAP patients are elevated [27]. Mortensen et al. [28], in a prospective cohort study with 1555 CAP patients, found that 8.7% of patients died within 90 days, and 30.3% died within 5 years of the acute presentation. Also, in a multicentric study of 3415 adults with CAP prospectively enrolled, Johnstone et al. [29] found that 30 day, 1 year, and 3.8 year mortality rates were 12%, 28%, and 53%, respectively. These studies are not specific to patients with CAP admitted to the ICU; however, these align with our results. We found a 6 m and 12 m mortality rate of 37.0% and 43.7%, respectively, demonstrating that mortality in patients with CAP admitted to the ICU is unacceptably high and undoubtedly increases the economic burden even after hospital discharge.

Several risk factors have been associated with higher mortality in patients with CAP. Regarding severity, the utility of the SAPS II score among long-term outcomes is still debatable [30, 31]. On top of that, other scores as the higher Charlson comorbidity Index, are associated with higher mortality during hospitalisation due to CAP [32]. Still, some medical conditions included in this score have been individually associated with worse long-term clinical outcomes. Almirall et al. [33], a systematic review confirmed that older age increases long-term fatal outcomes in CAP hospitalised patients. On the other hand, respiratory failure patients continue to have an increased mortality risk in the following months and years after the ICU discharge [34, 35]. Similarly, Wang et al. found that one-year mortality was significantly higher than in-hospital mortality in patients hospitalised with respiratory failure (41% vs 24%, *p* = 0.01) [36]. Finally, sepsis patients exhibited increased all-cause mortality rates up to 5 years after the acute infection [37]. This preliminary data aligns with our study's results.

Empiric antibiotic treatment has been described as the cornerstone of CAP management [13], and macrolide-based vs non-macrolide-based therapy is controversial in the literature [38–40]. One of the macrolides' benefits is to block bacterial toxins and have potential immunomodulatory properties that control disease progression [8, 20, 41]. However, Postma et al. [42] cluster-randomised crossover trial with CAP patients admitted to non-ICU wards concluded that non-macrolide-based treatment was a non-inferior strategy when analysing 90 day mortality. Nevertheless, Waterer et al. [38] identified problems with the methodology. 25% of the cohort had no radiological confirmation of pneumonia, and over one-third of patients in the monotherapy, β-lactam strategy received a macrolide antibiotic, resulting in an unbalanced intervention and a substantial risk of bias. König et al. used a multinational machine learning cross-validation scheme with 4898 [43]. They found that patients treated with non-macrolide-based treatment had a higher 180-day mortality than macrolide-based treatment [8.1% vs 7.6%; OR 1.06 (95% CI 0.82–1.36)]. A post hoc analysis of a cohort study of 594 CAP patients with low drug-resistant pathogen risk was performed by Okumura et al. [44] showed that those treated with macrolide treatment had better clinical outcomes regarding 30 day mortality [OR 0.28 (95% CI 0.09–0.87)]. Although these and other studies have demonstrated the acute benefit of macrolide-based treatment in patients with severe CAP, they have not assessed their long-term implications. Strikingly, our study is the first to identify a medication used during acute infection that could improve long-term outcomes, being novel and having important implications for clinical practice. To improve clinical outcomes, patients with CAP admitted to the ICU should be treated with macrolide-based antibiotic treatment. This therapy may also reduce long-term mortality and impact healthcare systems.

Our study has certain limitations that are important to acknowledge. First, this is a monocentric, observational, non-randomised study design. However, we included an extensive sample size of over three thousand patients over 10 years. Moreover, we conducted a TMLE (that simulates an RCT) to adjust results for potential confounding variables, controlling the risk of bias and enabling greater statistical power. Second, patients were enrolled in a high-income country, making it difficult to extrapolate and replicate the methodology to validate this data in low- and middle-income countries. However, clarithromycin is an inexpensive medication that could be used in limited-resource settings with myriad potential benefits. Third, no standardised protocols of antimicrobial treatment, doses, start time, and total days of administration were used, which also restricted the stratification analysis by these data. Nevertheless, macrolides are available globally and are used frequently in patients admitted to the hospital in the ICU with CAP using standard dosing. Also, the centres in this study used internationally accepted guidelines for using empirical antibiotics. Finally, we could not differentiate patients diagnosed with HCAP in our cohort. This might be a limitation because patients with HCAP were considered at risk of CAP due to *P. aeruginosa* and MRSA. Consequently, patients with HCAP were recommended to receive antipseudomonal and anti-MRSA coverage. However, no recommendation about macrolide usage was available for these patients; therefore, this classification may not interfere with our results. Also, we performed a sensitivity analysis excluding these patients and confirmed our results.

In conclusion, our study used a robust statistical analysis to demonstrate that macrolide-based treatment is associated with lower long-term mortality by reducing over one-third of the hazard risk; therefore, the benefit observed during acute hospitalisation is sustained over time. Thus, these data provide further justification for using macrolide-based treatment in patients with CAP admitted to the ICU to reduce the long-term burden of this prevalent disease. Additional prospective studies are required to support these conclusions.

## Supplementary Information

**Additional file 1: Table S1.** ICD-9 Codes. **Fig. S1.** Critical care invasive treatments and pneumonia causal agents. Panel A shows a Venn diagram of the different invasive treatments received. Panel B shows the most frequent causative microorganisms of pneumonia, being "other" specified in panel C. **Table S2.** Pneumonia casual agents. **Table S3.** Used antibiotics in the whole cohort and stratified between treatments. **Fig. S2.** Hosmer Lemeshow Test. Goodness of fit for logistic regression model was calculated, panel A shows result for six-months mortality and panel B for twelve-months mortality. **Fig. S3.** Propensity Score Matching. The original cohort is shown in panel A and the matched cohort is in panel B. **Fig. S4.** Logistic regression model to identify factors associated with 12 m mortality in the matched cohort. Logistic regression was performed with the optimal subset of variables obtained with the random forest model. The odds ratiosare graphically represented in the Forest plot for better medical interpretability. **Fig. S5.** Area under de Curve in the matched cohort. Cross-validation trial's receiver operative curvefor the subset of the selected variables. The blue curve represents the average of the ROC curves of each test, and the average area under de ROC is also presented. **Fig. S6.** Cox Proportional Hazard Regression to identify factors associated with 6 m and 12 m mortality. A Forest plot distribution of risk and protective factors for 6 m mortality in original cohort. B Forest plot distribution of risk and protective factors for 12 m mortality in original cohort. **Table S4.** Six-months mortality Cox Proportional Hazard Regression. **Table S5.** Twelve-months mortality Cox Proportional Hazard Regression. **Fig. S7.** Logistic regression model to identify factors associated with 6 m and 12 m mortality without *P. aeruginosa* or MRSA infected patients. Logistic regression was performed with the optimal subset of variables obtained with the random forest model. The odds ratiosare graphically represented in the Forest plot for better medical interpretability. Panel A has presented the odd proportions of the risk for 6 m mortality, and panel B is shown for 12 m mortality. **Fig. S8.** Area under de Curve without *P. aeruginosa* or MRSA infected patients. Cross-validation trial's receiver operative curvefor the subset of the selected variables. The blue curve represents the average of the ROC curves of each test, and the average area under de ROC is also presented. Panel A presents the AUC-ROC for 6 m mortality and panel B for 12 m mortality.

## Data Availability

The full dataset could be shared by direct request to the corresponding author.
