# Supplementary material for: Impact of macrolide treatment on long-term mortality in patients admitted to the ICU due to CAP: a targeted maximum likelihood estimation and survival analysis
Source: Crit Care. 2023 May 31;27:212. doi: 10.1186/s13054-023-04466-x (PMC10230128; doi:10.1186/s13054-023-04466-x)
Supplement: Supplementary file 1 — Additional file 1: Table S1. ICD-9 Codes. Fig. S1. Critical care invasive treatments and pneumonia causal agents. Panel A shows a Venn diagram of the different invasive treatments received. Panel B shows the most frequent causative microorganisms of pneumonia, being "other" specified in panel C. Table S2. Pneumonia casual agents. Table S3. Used antibiotics in the whole cohort and stratified between treatments. Fig. S2. Hosmer Lemeshow Test. Goodness of fit for logistic regression model was calculated, panel A shows result for six-months mortality and panel B for twelve-months mortality. Fig. S3. Propensity Score Matching. The original cohort is shown in panel A and the matched cohort is in panel B. Fig. S4. Logistic regression model to identify factors associated with 12 m mortality in the matched cohort. Logistic regression was performed with the optimal subset of variables obtained with the random forest model. The odds ratiosare graphically represented in the Forest plot for better medical interpretability. Fig. S5. Area under de Curve in the matched cohort. Cross-validation trial's receiver operative curvefor the subset of the selected variables. The blue curve represents the average of the ROC curves of each test, and the average area under de ROC is also presented. Fig. S6. Cox Proportional Hazard Regression to identify factors associated with 6 m and 12 m mortality. A Forest plot distribution of risk and protective factors for 6 m mortality in original cohort. B Forest plot distribution of risk and protective factors for 12 m mortality in original cohort. Table S4. Six-months mortality Cox Proportional Hazard Regression. Table S5. Twelve-months mortality Cox Proportional Hazard Regression. Fig. S7. Logistic regression model to identify factors associated with 6 m and 12 m mortality without P. aeruginosa or MRSA infected patients. Logistic regression was performed with the optimal subset of variables obtained with the random forest model. The odds ratiosare gr [file 13054_2023_4466_MOESM1_ESM.docx]

**TITLE:** Impact of macrolide treatment on long-term mortality in patients admitted to the ICU due to CAP: A targeted maximum likelihood estimation and survival analysis.

**AUTHORS:** Luis Felipe Reyes^1, 2, 3*#^, Esteban Garcia^1*^, Elsa D. Ibáñez-Prada^2^, Cristian C. Serrano-Mayorga^2^, Yuli V. Fuentes^1, 2^, Alejandro Rodríguez^4^, Gerard Moreno^4^, Alirio Bastidas^1^, Josep Gómez^4^, Angélica Gonzalez^1^, Christopher R Frei^5, 6^, Leo Anthony Celi^7,8^ Ignacio Martin-Loeches^10^ and Grant Waterer^11^.

*Co-first authors

**AFFILIATIONS:** 1, Universidad de La Sabana, Chía, Colombia; 2, Clínica Universidad de La Sabana; 3, University of Oxford, Oxford, United Kingdom; 4, Hospital Universitari Joan XXIII, Critical Care Medicine, Rovira and Virgili University and CIBERES (Biomedical Research Network of Respiratory Disease), Tarragona, Spain; 5, College of Pharmacy, The University of Texas at Austin, San Antonio, Texas, USA; 6, School of Medicine, University of Texas Health San Antonio, San Antonio, Texas, USA; 7, Massachusetts Institute of Technology, USA; 8, Beth Israel Deaconess Medical Center, USA; 9, Harvard T.H. Chan School of Public Health, USA; 10, Department of Intensive Care Medicine, Multidisciplinary Intensive Care Research Organisation (MICRO), St. James's Hospital, Dublin; 11, Royal Perth Bentley Hospital Group, University of Western Australia, Perth, Australia.

**Corresponding author^#^:** Luis Felipe Reyes, MD, PhD.; Universidad de La Sabana, Campus Puente del Común, KM 7.5 Autopista Norte de Bogotá, Chía, Colombia. Phone: (571) 861-5555 ext. 23342; Email: [luis.reyes5@unisabana.edu.co](mailto:luis.reyes5@unisabana.edu.co)

**FIGURES**

**Figure S1. Critical care invasive treatments and pneumonia causal agents.** Panel A shows a Venn diagram of the different invasive treatments received. Panel B shows the most frequent causative microorganisms of pneumonia, being "other" specified in panel C.

**Figure S2. Hosmer Lemeshow** **Test**. Goodness of fit for logistic regression model was calculated, panel A shows result for six-months mortality and panel B for twelve-months mortality.

**Figure S3. Propensity Score Matching (PSM).** The original cohort is shown in panel A and the matched cohort is in panel B.

**Figure S4. Logistic regression model to identify factors associated with 12m mortality in the matched cohort.** Logistic regression was performed with the optimal subset of variables obtained with the random forest model. The odds ratios (OR) are graphically represented in the Forest plot for better medical interpretability.

**Figure S5. Area Under de Curve in the matched cohort.** Cross-validation trial's receiver operative curve (ROC) for the subset of the selected variables. The blue curve represents the average of the ROC curves of each test, and the average area under de ROC is also presented.

**Figure S6. Cox Proportional Hazard Regression to identify factors associated with 6m and 12m mortality.** (A) Forest plot distribution of risk and protective factors for 6m mortality in original cohort. (B) Forest plot distribution of risk and protective factors for 12m mortality in original cohort.

**Figure S7. Logistic regression model to identify factors associated with 6m and 12m mortality without *P. aeruginosa* or MRSA infected patients.** Logistic regression was performed with the optimal subset of variables obtained with the random forest model. The odds ratios (OR) are graphically represented in the Forest plot for better medical interpretability. Panel A has presented the odd proportions of the risk for 6m mortality, and panel B is shown for 12m mortality.

**Figure S8. Area Under de Curve without *P. aeruginosa* or MRSA infected patients.** Cross-validation trial's receiver operative curve (ROC) for the subset of the selected variables. The blue curve represents the average of the ROC curves of each test, and the average area under de ROC is also presented. Panel A presents the AUC-ROC for 6m mortality and panel B for 12m mortality.

**FIGURES**

**Figure S1. Critical care invasive treatments and pneumonia causal agents.**

**Figure S2. Hosmer Lemeshow** **Test**

**Figure S3. Propensity Score Matching (PSM).**

**Figure S4. Logistic regression model to identify factors associated with 12m mortality in the matched cohort.**

**Figure S5. Area Under de Curve in the matched cohort.**

**
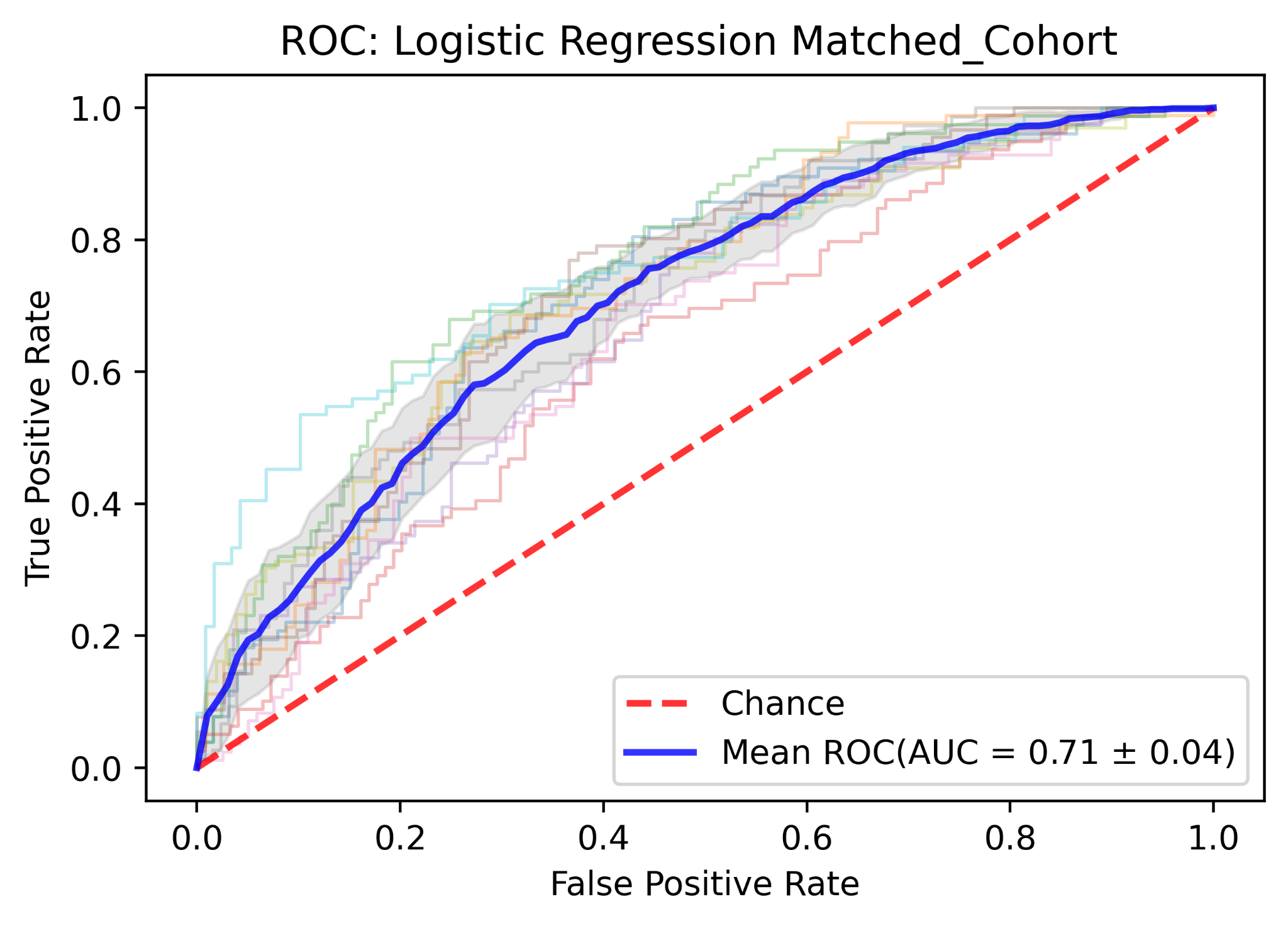
**

**Figure S6. Cox Proportional Hazard Regression to identify factors associated with 6m and 12m mortality.**

**Figure S7. Logistic regression model to identify factors associated with 6m and 12m mortality without *P. aeruginosa* or MRSA -infected patients.**

**Figure S8. Area Under de Curve without *P. aeruginosa* or MRSA-infected patients.**

**TABLES**

**Table S1.** ICD-9 Codes

| **Index** | **Frequency** |
| --- | --- |
| Pneumonia, organism unspecified | 1518 |
| Pneumonia, unspecified organism | 1007 |
| Bacterial pneumonia, unspecified | 173 |
| Pneumonia due to Pseudomonas | 151 |
| Unspecified bacterial pneumonia | 110 |
| Methicillin resistant pneumonia due to Staphylococcus aureus | 98 |
| Methicillin susceptible pneumonia due to Staphylococcus aureus | 84 |
| Pneumonia due to Klebsiella pneumoniae | 71 |
| Pneumonia due to Methicillin susceptible Staphylococcus aureus | 65 |
| Pneumonia due to other gram-negative bacteria | 57 |
| Pneumonia due to other Gram-negative bacteria | 55 |
| Pneumococcal pneumonia [Streptococcus pneumoniae pneumonia] | 55 |
| Pneumonia due to Methicillin resistant Staphylococcus aureus | 43 |
| Pneumonia due to Streptococcus pneumoniae | 42 |
| Other pneumonia, unspecified organism | 38 |
| Pneumonia due to Hemophilus influenzae [H. influenzae] | 36 |
| Influenza with pneumonia | 35 |
| Pneumococcal septicemia [Streptococcus pneumoniae septicemia] | 32 |
| Pneumonia due to Hemophilus influenzae | 26 |
| Influenza due to other identified influenza virus with other specified pneumonia | 23 |
| Pneumonia due to escherichia coli [E. coli] | 20 |
| Pneumonia due to other specified bacteria | 20 |
| Pneumonia due to Escherichia coli | 18 |
| Abscess of lung with pneumonia | 18 |
| Sepsis due to Streptococcus pneumoniae | 17 |
| Lobar pneumonia, unspecified organism | 13 |
| Influenza due to other identified influenza virus with unspecified type of pneumonia | 12 |
| Influenza with other respiratory manifestations | 11 |
| Pneumonia due to Legionnaires' disease | 10 |
| Influenza due to unidentified influenza virus with unspecified type of pneumonia | 9 |
| Pneumonia due to other Streptococcus | 8 |
| Pneumonia due to mycoplasma pneumoniae | 7 |
| Influenza due to unidentified influenza virus with specified pneumonia | 7 |
| Bronchopneumonia, organism unspecified | 7 |
| Influenza due to identified novel influenza A virus with pneumonia | 6 |
| Klebsiella pneumoniae [K. pneumoniae] as the cause of diseases classified elsewhere | 5 |
| Pneumonia due to other specified organism | 5 |
| Influenza due to identified avian influenza virus with pneumonia | 5 |
| Pneumonia due to Staphylococcus, unspecified | 5 |
| Influenza due to other identified influenza virus with other respiratory manifestations | 4 |
| Septicemia due to hemophilus influenzae [H. influenzae] | 4 |
| Hemophilus influenzae [H. influenzae] infection in conditions classified elsewhere and of unspecified site | 4 |
| Influenza due to identified novel influenza A virus with other respiratory manifestations | 4 |
| Influenza due to other identified influenza virus with the same other identified influenza virus pneumonia | 4 |
| Pneumonia due to streptococcus, group B | 3 |
| Sepsis due to Hemophilus influenzae | 3 |
| Pneumonia due to Mycoplasma pneumoniae | 3 |
| Pneumonia due to Streptococcus, group B | 3 |
| Pneumonia due to other streptococci | 3 |
| Other Staphylococcus pneumonia | 3 |
| Pneumonia due to Streptococcus unspecified | 3 |
| Influenza due to identified novel H1N1 influenza virus | 2 |
| Influenza due to unidentified influenza virus with other respiratory manifestations | 2 |
| Pneumonia due to other staphylococcus | 2 |
| Pneumonia in diseases classified elsewhere | 1 |
| Streptococcus pneumoniae as the cause of diseases classified elsewhere | 1 |
| Influenza due to identified 2009 H1N1 influenza virus with pneumonia | 1 |
| Pneumonia due to staphylococcus, unspecified | 1 |
| Pneumonia due to Streptococcus group A | 1 |
| Hemophilus influenzae [H. influenzae] as the cause of diseases classified elsewhere | 1 |
| Pneumonia due to other specified infectious organisms | 1 |

**Table S2.** Pneumonia casual agents.

| **Microorganism** | **All**  **(n=3775)** | **Macrolide-based (n=1154)** | **Non-Macrolide-based (n=2621)** |
| --- | --- | --- | --- |
| *Non-Identified* | 2648 (70.1) | 947 (82.1) | 1701 (64.9) |
| *S. aureus* | 383 (10.1) | 65 (5.6) | 318 (12.1) |
| *P. aeruginosa* | 176 (4.7) | 33 (2.9) | 143 (5.5) |
| *K. pneumoniae* | 98 (2.6) | 9 (0.8) | 89 (3.4) |
| *S. pneumoniae* | 69 (1.8) | 20 (1.7) | 49 (1.9) |
| *E. coli* | 68 (1.8) | 10 (0.9) | 58 (2.2) |
| *S. maltophila* | 59 (1.6) | 14 (1.2) | 45 (1.7) |
| *S. marcescens* | 38 (1.0) | 4 (0.3) | 34 (1.3) |
| *Aspergillus sp.* | 37 (1.0) | 16 (1.4) | 21 (0.8) |
| *M. catarrhalis* | 34 (0.9) | 8 (0.7) | 26 (1.0) |
| *A. baumannii* | 32 (0.8) | 4 (0.3) | 28 (1.1) |
| *E. cloacae* | 31 (0.8) | 1 (0.1) | 30 (1.1) |
| *E. aerogenes* | 21 (0.6) | 3 (0.3) | 18 (0.7) |
| *K. oxytoca* | 13 (0.3) | 2 (0.2) | 11 (0.4) |
| *Corynebacterium sp.* | 11 (0.3) | 4 (0.3) | 7 (0.3) |
| *C. freundii* | 6 (0.2) | 0 (0.0) | 6 (0.2) |
| *E. asburiae* | 6 (0.2) | 2 (0.2) | 4 (0.2) |
| *B. cepacia* | 5 (0.1) | 1 (0.1) | 4 (0.2) |
| *C. koseri* | 5 (0.1) | 3 (0.3) | 2 (0.1) |
| *M. morganii* | 4 (0.1) | 0 (0.0) | 4 (0.2) |
| *Acinetobacter sp.* | 3 (0.1) | 1 (0.1) | 2 (0.1) |
| *H. alvei* | 3 (0.1) | 0 (0.0) | 3 (0.1) |
| *Haemophilus sp.* | 3 (0.1) | 1 (0.1) | 2 (0.1) |
| *P. jirovecii* | 3 (0.1) | 2 (0.2) | 1 (0.0) |
| *P. stuartii* | 3 (0.1) | 0 (0.0) | 3 (0.1) |
| *Alcaligenes sp.* | 2 (0.1) | 1 (0.1) | 1 (0.0) |
| *H. influenzae* | 2 (0.1) | 0 (0.0) | 2 (0.1) |
| *M. tuberculosis* | 2 (0.1) | 1 (0.1) | 1 (0.0) |
| *S. anginosus* | 2 (0.1) | 0 (0.0) | 2 (0.1) |
| *A. hydrophila* | 1 (0.0) | 0 (0.0) | 1 (0.0) |
| *Adenovirus* | 1 (0.0) | 0 (0.0) | 1 (0.0) |
| *L. pneumophila* | 1 (0.0) | 0 (0.0) | 1 (0.0) |
| *P. stutzeri* | 1 (0.0) | 0 (0.0) | 1 (0.0) |
| *S. liquefaciens* | 1 (0.0) | 0 (0.0) | 1 (0.0) |
| *Serratia sp.* | 1 (0.0) | 0 (0.0) | 1 (0.0) |
| *Respiratory syncytial virus* | 1 (0.0) | 1 (0.1) | 0 (0.0) |
| *S. rubidaea* | 1 (0.0) | 1 (0.1) | 0 (0.0) |

**Table S3**. Used antibiotics in the whole cohort and stratified between treatments.

| **Antibiotic** | **All**  **(n=3775)** | **Macrolide-based (n=1154)** | **Non-Macrolide-based (n=2621)** |
| --- | --- | --- | --- |
| Vancomycin | 1809 (47.9) | 471 (40.8) | 1338 (51.0) |
| Cefepime | 1001 (26.5) | 291 (25.2) | 710 (27.1) |
| Macrolide | 974 (25.8) | 974 (84.4) | 0 (0.0) |
| Levofloxacin | 692 (18.3) | 39 (3.4) | 653 (24.9) |
| Ceftriaxone | 618 (16.4) | 433 (37.5) | 185 (7.1) |
| Piperacillin/Tazobactam | 518 (13.7) | 113 (9.8) | 405 (15.5) |
| Meropenem | 125 (3.3) | 30 (2.6) | 95 (3.6) |
| Ceftazidime | 115 (3.0) | 47 (4.1) | 68 (2.6) |
| Ciprofloxacin | 93 (2.5) | 12 (1.0) | 81 (3.1) |
| Cefazolin | 66 (1.7) | 8 (0.7) | 58 (2.2) |
| Doxycycline | 60 (1.6) | 3 (0.3) | 57 (2.2) |
| Linezolid | 44 (1.2) | 8 (0.7) | 36 (1.4) |
| Ampicillin/Sulbactam | 37 (1.0) | 2 (0.2) | 35 (1.3) |
| Clindamycin | 23 (0.6) | 2 (0.2) | 21 (0.8) |
| Cefpodoxime | 15 (0.4) | 11 (1.0) | 4 (0.2) |
| Amoxicillin/Clavulanic Acid | 12 (0.3) | 2 (0.2) | 10 (0.4) |
| Ampicilin | 11 (0.3) | 3 (0.3) | 8 (0.3) |
| Amikacin | 6 (0.2) | 1 (0.1) | 5 (0.2) |
| Gentamicin | 5 (0.1) | 0 (0.0) | 5 (0.2) |
| Imipenem/Cilastatin | 5 (0.1) | 2 (0.2) | 3 (0.1) |
| Penicillin | 4 (0.1) | 0 (0.0) | 4 (0.2) |
| Cephalexin | 2 (0.1) | 2 (0.2) | 0 (0.2) |
| Amoxacilin | 1 (0.0) | 0 (0.0) | 1 (0.0) |

**Table S4**. Six-months mortality Cox Proportional Hazard Regression

| **Covariate** | **HR** | **95% CI** |
| --- | --- | --- |
| ARDS | 1.92 | (1.20 - 3.06) |
| Septic Shock | 1.57 | (1.38 - 1.77) |
| Respiratory Failure | 1.46 | (1.28 - 1.66) |
| No aetiology | 1.23 | (1.08 - 1.40) |
| INR max | 1.21 | (0.93 - 1.58) |
| Male | 1.18 | (1.06 - 1.33) |
| Charlson Comorbidity Index | 1.12 | (1.10 - 1.15) |
| HFNC | 1.09 | (0.80 - 1.48) |
| WBC min | 1.02 | (1.01 - 1.04) |
| SAPS II | 1.02 | (1.01 - 1.02) |
| Calcium max | 1.02 | (0.96 - 1.08) |
| Bicarbonate min | 1.02 | (1.00 - 1.03) |
| Angion gap max | 1.01 | (0.99 - 1.03) |
| Haematocrit min | 1.01 | (0.99 - 1.02) |
| Age | 1.01 | (1.00 - 1.01) |
| Lymphocytes max | 1.00 | (0.99 - 1.01) |
| BUN max | 1.00 | (1.00 - 1.01) |
| PTT max | 1.00 | (1.00 - 1.00) |
| Neutrophils max | 1.00 | (0.99 - 1.01) |
| Platelets min | 1.00 | (1.00 - 1.00) |
| Urine Output | 1.00 | (1.00 - 1.00) |
| Glucose min | 1.00 | (1.00 - 1.00) |
| Typical Bacteria | 1.00 | (0.79 - 1.27) |
| Sodium max | 1.00 | (0.98 - 1.01) |
| Choride min | 1.00 | (0.98 - 1.01) |
| *P. aeruginosa* or MRSA | 0.99 | (0.85 - 1.15) |
| PT max | 0.99 | (0.96 - 1.01) |
| WBC max | 0.98 | (0.97 - 1.00) |
| Atypical bacteria | 0.98 | (0.81 - 1.19) |
| Potassium max | 0.97 | (0.91 - 1.03) |
| Haemoglobin max | 0.92 | (0.88 - 0.96) |
| Fungi | 0.90 | (0.55 - 1.45) |
| Invasive Mechanical Ventilation | 0.85 | (0.74 - 0.97) |
| Quinolone treatment | 0.84 | (0.72 - 0.98) |
| Non-Invasive Mechanical Ventilation | 0.84 | (0.64 - 1.10) |
| Creatinine min | 0.83 | (0.78 - 0.88) |
| Macrolide treatment | 0.69 | (0.60 - 0.78) |
| ARDS: Acute respiratory distress syndrome; INR: International Normalized Ratio; HFNC: High Flow Nasal Cannula; WBC: white blood cells; SAPS II: Simplified Acute Physiology Score; BUN: Blood Urea Nitrogen; PT: Prothrombin Time; PTT: Partial Thromboplastin Time. | | |

**Table S5**. Twelve-months mortality Cox Proportional Hazard Regression

| **Covariate** | **HR** | **95% CI** |
| --- | --- | --- |
| ARDS | 1.75 | (1.11 - 2.76) |
| Septic Shock | 1.51 | (1.34 - 1.69) |
| Respiratory Failure | 1.35 | (1.20 - 1.52) |
| INR max | 1.21 | (0.96 - 1.53) |
| No aetiology | 1.18 | (1.05 - 1.33) |
| Male | 1.17 | (1.06 - 1.30) |
| Charlson Comorbidity Index | 1.13 | (1.11 - 1.15) |
| WBC min | 1.02 | (1.01 - 1.04) |
| HFNC | 1.02 | (0.77 - 1.36) |
| Bicarbonate min | 1.02 | (1.00 - 1.03) |
| SAPS II | 1.02 | (1.01 - 1.02) |
| Calcium max | 1.02 | (0.96 - 1.07) |
| Angion gap max | 1.01 | (0.99 - 1.03) |
| Haematocrit min | 1.01 | (0.99 - 1.02) |
| Age | 1.01 | (1.00 - 1.01) |
| Lymphocytes max | 1.00 | (0.99 - 1.02) |
| Atypical bacteria | 1.00 | (0.83 - 1.21) |
| BUN max | 1.00 | (1.00 - 1.01) |
| PTT max | 1.00 | (1.00 - 1.00) |
| Neutrophils max | 1.00 | (0.99 - 1.01) |
| Sodium max | 1.00 | (0.99 - 1.01) |
| Urine Output | 1.00 | (1.00 - 1.00) |
| Platelets min | 1.00 | (1.00 - 1.00) |
| P. aeruginosa or MRSA | 1.00 | (0.86 - 1.16) |
| Glucose min | 1.00 | (1.00 - 1.00) |
| Chloride min | 0.99 | (0.98 - 1.01) |
| Potassium max | 0.99 | (0.94 - 1.05) |
| PT max | 0.99 | (0.96 - 1.01) |
| WBC max | 0.98 | (0.97 - 1.00) |
| Typical bacteria | 0.98 | (0.78 - 1.23) |
| Haemoglobin max | 0.91 | (0.87 - 0.96) |
| Fungi | 0.87 | (0.55 - 1.38) |
| Quinolone treatment | 0.86 | (0.75 - 0.99) |
| Invasive Mechanical Ventilation | 0.85 | (0.75 - 0.96) |
| Creatinine min | 0.84 | (0.79 - 0.89) |
| Non-Invasive Mechanical Ventilation | 0.82 | (0.64 - 1.06) |
| Macrolide treatment | 0.72 | (0.64 - 0.81) |
| ARDS: Acute respiratory distress syndrome; INR: International Normalized Ratio; HFNC: High Flow Nasal Cannula; WBC: white blood cells; SAPS II: Simplified Acute Physiology Score; BUN: Blood Urea Nitrogen; PT: Prothrombin Time; PTT: Partial Thromboplastin Time. | | |
